# Robotics versus personalized 3D preoperative planning in total knee arthroplasty: a propensity score-matched analysis

**DOI:** 10.1186/s13018-022-03115-3

**Published:** 2022-04-11

**Authors:** Kai Lei, LiMing Liu, PengFei Yang, Ran Xiong, Liu Yang, Rui He, Lin Guo

**Affiliations:** Center for Joint Surgery, Southwest Hospital, Third Military Medical University, No. 30 Gaotanyan Street, Shapingba District, Chongqing, 400036 China

**Keywords:** Total knee arthroplasty, Alignment, Prosthesis positioning, Robotics, Personalized 3D preoperative planning, Propensity score matching

## Abstract

**Purpose:**

Lower limb alignment is crucial in total knee arthroplasty (TKA). Previous studies have shown that robotics and personalized three-dimensional (3D) preoperative planning could improve postoperative alignment accuracy compared with conventional TKA, but comparison between the above two techniques has never been reported. The authors hypothesized that robotics may be superior to personalized 3D preoperative planning in terms of postoperative alignment in primary TKA, with similar patient-reported outcome measures (PROMs) but higher cost and longer operative time.

**Methods:**

A consecutive series of patients who received TKA in our center from September 2020 to January 2021 were enrolled retrospectively. After 1:2 matching, 52 and 104 patients were included and divided into study group for robotics and control group for personalized 3D preoperative planning, respectively. Multiple postoperative alignment angles were measured, and clinical features such as operation and tourniquet time, length of hospital stay and hemoglobin (Hb) were recorded. Knee Society Score (KSS) and Western Ontario and McMaster Universities Osteoarthritis Index (WOMAC) were used to evaluate clinical results.

**Results:**

Compared with control group, robotics group had significantly lower frontal femoral component angle (FFC) and frontal tibial component angle (FTC) absolute deviation (*P* < 0.05). It also had less outliers in hip–knee–ankle angle (HKA), FTC, lateral femoral component angle (LFC) and lateral tibial component angle (LTC) (*P* < 0.05). Hb loss of robotics group was significantly lower than control group (*P* < 0.001), while the operation and tourniquet time were longer (*P* < 0.001). There was no significant difference in KSS and WOMAC scores between two groups.

**Conclusion:**

Compared with control group, patients in robotics group had significantly less malalignment, malposition, Hb loss, but similar PROMs. The operations in robotics group spent longer operation time and cost more compared with control group.

*Trial registration*: The Chinese Clinical Trial Registry, ChiCTR2000036235. Registered 22 August 2020, http://www.chictr.org.cn/showproj.aspx?proj=59300.

**Level of evidence:**

III.

**Supplementary Information:**

The online version contains supplementary material available at 10.1186/s13018-022-03115-3.

## Introduction

Substantial studies have demonstrated that accurate alignment and prosthesis positioning in total knee arthroplasty (TKA) are closely related to satisfactory postoperative outcomes and prosthesis longevity [1–5]. Although contemporary prosthesis designs have enhanced durability, the longer life expectancies of patients put higher demands on prosthesis survivorship [6]. In addition, postoperative dissatisfaction following TKA is still high up to 20% [7, 8]. TKA is one of the most effective interventions for end-stage knee osteoarthritis, while improvements in surgical technique remain to be necessity [9]. In order to improve alignment and prosthesis positioning accuracy, thereby reducing revision and improving outcomes, some advanced techniques have been adopted such as computer navigation [10], patient-specific instrumentation (PSI) [11] and robot-assisted surgery [12].

The alignment and prosthesis positioning accuracy among navigation, PSI, robotics and conventional TKA were compared in the previous large sample meta-analysis [13, 14]. A Bayesian network meta-analysis included a total of 73 randomized controlled trials (RCTs) involving 4209 TKAs and found that robotics significantly reduced the occurrence of malalignment and malposition compared with conventional TKA [13]. However, increased medical costs and longer operation time greatly limit the application of robotics during TKA [9, 15, 16].

To achieve accurate alignment and prosthesis positioning with a cost-effective method, we reported previously a verified technique which used personalized 3D preoperative planning [17]. It could be considered as a simplified PSI without 3D-printed cutting guides. Multiple bone markers such as femoral entry point and resting point of the pin of tibial extramedullary cutting guide were used for positioning of conventional resection instruments rather than patient-specific cutting guides. Advantages of this technique include personalized preoperative planning, precise intraoperative positioning, no need for new equipment, better control of the surgical time and cost, and easier learning curve due to similar procedures like conventional TKA [17].

The previous study confirmed that personalized 3D preoperative planning could improve resection accuracy compared with conventional TKA [17], while to our knowledge the comparison between robotics and personalized 3D preoperative planning has never been reported. The purpose of this study was to investigate the above issues. The authors hypothesized that robotics may be superior to personalized 3D preoperative planning in terms of postoperative alignment in primary TKA, with similar patient-reported outcome measures (PROMs) but higher cost and longer operative time.

## Materials and methods

Medical records and imaging data were retrospectively collected from a consecutive series of TKA performed in our center from September 2020 to January 2021. The robotics group included patients with knee osteoarthritis, who underwent TKA with the Skywalker™ robotics system (MicroPort® OrthoBot Co., Ltd., Suzhou, China); the 3D preoperative planning group included patients with knee osteoarthritis who underwent TKA with personalized 3D preoperative planning. Patients failed to achieve pre- and postoperative full-length weight-bearing radiographs (FLX) or did not meet Paley’s criteria [18] were excluded. 52 cases were enrolled in robotics group and 196 cases were enrolled in 3D preoperative planning control group.

Previous literatures indicated that malalignment rates of robotics and 3D preoperative planning after TKA was about 2% and 18%, respectively [12, 17, 19, 20]. The power level was set at 0.95 with two-sided α at 0.05 in this study (1 − *β* = 0.95, *α* = 0.05). The power analysis by PASS 15.0 revealed that 48 in robotics group and 96 in 3D preoperative planning group would be an appropriate sample size. To reduce the influence of selection bias and potential confounding factors in this retrospective study, the gender, left or right, age, body mass index (BMI) and preoperative hip–knee–ankle angle (HKA) were selected to perform a 1:2 matching with the “nearest” method by R software (Version 4.0.4, R foundation for statistical Computing, Vienna, Austria). Finally, 52 robot-assisted TKAs and 104 personalized 3D preoperative planning TKAs were compared in this study.

The personalized 3D preoperative planning [17] and Legion® primary total knee prosthesis (Smith-Nephew, Inc., Memphis, IN, USA) were used in the 3D preoperative planning group. The lower limb full-length computed tomography (CT) data of patients was collected to perform 3D reconstruction with Mimics Research 19.0. With the CATIA 5.20 and NX12.0 software, the engineers and surgeons formulated the personalized 3D preoperative planning, which should include the following key information: the femoral entry point, the coronal projection angle of the hip–knee–shaft (HKS), the transverse projection angle of the posterior condylar angle (PCA), the fix point of the tibial plateau extramedullary guide pin, the volume of femoral and tibial resection, etc. [17]. During the intraoperative implementation, the femoral entry point was strictly located according to the preoperative plan, and the coronal projection angle of HKS and the specific resection volume were used in the distal femoral resection. And the femoral rotatory resection was guided by the transverse projection angle of PCA. Similarly, the tibial resection was conducted based on the key information presented in the personalized 3D preoperative planning, including the fix point of the tibial plateau extramedullary guide pin, the tibial resection volume, etc. [17]. Please refer to Additional file 1* o*r the previous article [17] for more details on personalized 3D preoperative planning.

The Skywalker™ robotics system and Advance® medial-pivot knee prosthesis (MicroPort Orthopedics Inc., Arlington, TN, USA) were used in robotics group. A patient-specific 3D model was formulated automatically after importing the patients' lower limb CT data into the Skywalker™ robotics system. Multiple feature points were marked in the 3D model, such as the center point of femoral head, knee joint and ankle joint, the most prominent point of lateral femoral epicondyle, and the most concave point of medial femoral epicondyle. Then, the appropriate prosthesis positioning and alignment parameters were selected in preview to form a preoperative planning. During the surgery, the navigation markers made by radix lens (retroreflective lens for optical measurement) were installed and the patient's anatomical characteristics were registered to fit the preoperative plan. Under the help of optical measurement technology, the robotic arm with a cutting guide at the distal end automatically moved to the appropriate position and assisted the surgeons to complete accurate resection with saw.

In both groups, nerve block anesthesia and medial parapatellar approach were conducted. A tourniquet was applied before skin incision and released after the closure of joint capsule. In order to reduce total blood loss, tranexamic acid was routinely used. No patella replacement was performed, and all cases were conducted following mechanical alignment. Discharge criteria included that there were no obvious swelling, no extension lag, active bending ≥ 90°, walking distance with assistance ≥ 200 m and VAS pain score ≤ 4. Patients who met all the above criteria could be discharged. The hospital stay included postoperative rehabilitation programs education.

**Fig. 1:**
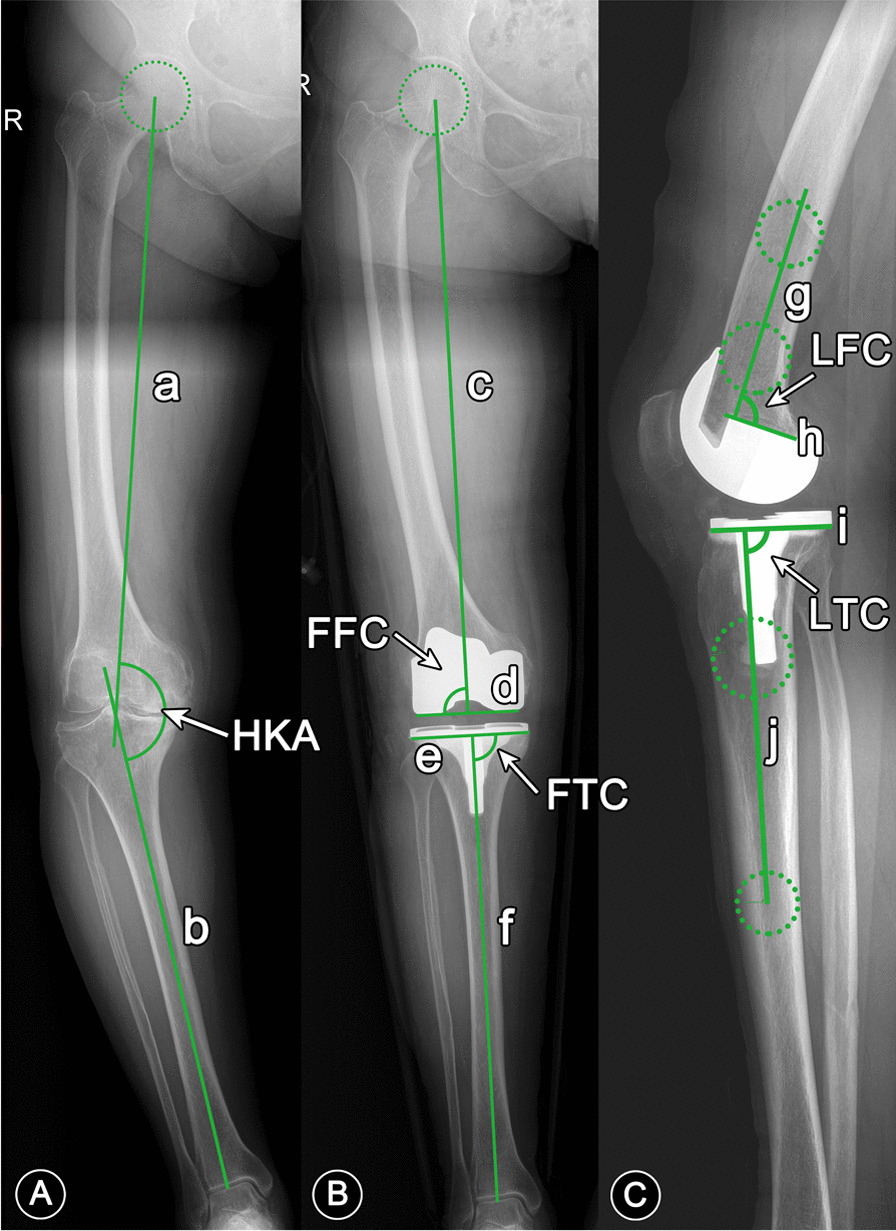
Measurement of HKA, FFC, FTC, LFC and LTC. HKA, hip–knee–ankle angle; FFC, frontal femoral component angle; FTC, frontal tibial component angle; LFC, lateral femoral component angle; and LTC, lateral tibial component angle

Preoperative FLX (within 1 month before surgery) and FLX of the latest follow-up were collected. Preoperative HKA and postoperative frontal femoral component (FFC) angle, frontal tibial component (FTC) angle, lateral femoral component (LFC) angle, lateral tibial component (LTC) angle and HKA (Fig. 1) were measured for three times by two raters independently [17], with an interval of more than 15 days. The targets in both groups for postoperative HKA, FFC, FTC, LFC and LTC were 180°, 90°, 90°, 90° and 87°, respectively. Values exceeding the target by 3 degrees were recorded as outliers. Medical records such as gender (male or female), side (left or right), age (years), BMI (kg/m^2^), operation time (min), tourniquet time (min), hospital stay (day) and hemoglobin (Hb) loss at 1 and 3 days after operation (g/L) were collected via the electronic medical record management system. Meanwhile, KSS and WOMAC scores were obtained preoperatively and 3, 6, 12 months after operation during outpatient follow-ups. Based on the guidance [21], WOMAC scores were standardized, ranging from 0 (worst) to 100 (best).

The *χ*^2^ test and *T* test was respectively used for categorical and continuous variables. Intraclass correlation coefficient (ICC) was used to evaluate intra-rater and inter-rater consistency in FLX measurement. ICC values less than 0.5, between 0.5 and 0.75, between 0.75 and 0.9, and greater than 0.90 were indicative of poor, moderate, good and excellent reproducibility, respectively [22]. Statistical analysis was performed by SPSS 25.0 (SPSS Inc., Chicago, IL), and *P* < 0.05 was considered statistically significant.

## Results

All baseline characteristics were similar between two groups after 1:2 matching (Table 1).

**Table 1:** Baseline characteristics before and after 1:2 matching

Characteristics	Before PSM (*n* = 248)			After PSM (*n* = 156)		
	3D preoperative planning group (*n* = 196)	Robotics group (*n* = 52)	*P *value	3D preoperative planning group (*n* = 104)	Robotics group (*n* = 52)	*P *value
Gender (male/female)	52:144	11:41	0.428^b^	23:81	11:41	0.891^b^
Side (left/right)	95:101	31:21	0.153^b^	61:43	31:21	0.908^b^
Age (years)	69.3 ± 8.4	66.1 ± 7.9	**0.016**^a^	66.9 ± 9.4	66.1 ± 7.9	0.585^a^
BMI (kg/m^2^)	25.6 ± 3.2	26.0 ± 4.2	0.472^a^	26.0 ± 3.5	26.0 ± 4.2	0.988^a^
Pre-HKA (°)	170.6 ± 8.8	172.4 ± 6.4	0.110^a^	172.1 ± 9.1	172.4 ± 6.4	0.857^a^

PSM, propensity score matching; BMI, body mass index; and HKA, hip–knee–ankle angle

^a^Stands for *t* test, and ^b^Stands for *χ*^2^ test. *P* value in bold indicates statistical significance

The intra-rater and inter-rater consistency in FLX measurement was excellent (ICC > 0.9, *P* < 0.05). The postoperative HKA and FFC were significantly different between two groups (*P* < 0.05), while the two groups had similar postoperative FTC, LFC and LTC. The two groups had similar absolute deviations from the target value in HKA, LFC and LTC, but the robotics group was significantly better in FFC and FTC absolute deviation (*P* < 0.05). Besides, the robotics group had significantly less outliers compared with the 3D preoperative planning group in HKA, FTC, LFC and LTC (*P* < 0.05) (Table 2).

**Table 2:** Comparison of postoperative alignment

	3D preoperative planning group (*n* = 104)	Robotics group (*n* = 52)	*P *value
HKA	179.3° ± 2.5°	181.1° ± 1.6°	**< 0.001**^a^
FFC	90.3° ± 1.9°	89.5° ± 1.5°	**0.007**^a^
FTC	89.5° ± 2.1°	90.0° ± 1.3°	0.077^a^
LFC	88.2° ± 2.5°	88.5° ± 2.3°	0.577^**a**^
LTC	86.0° ± 2.7°	86.7° ± 2.3°	0.100^a^
HKA absolute deviation	2.0° ± 1.7°	1.7° ± 1.0°	0.150^a^
FFC absolute deviation	1.6° ± 1.0°	1.3° ± 0.9°	**0.039**^a^
FTC absolute deviation	1.7° ± 1.3°	1.0° ± 0.7°	**< 0.001**^a^
LFC absolute deviation	2.6° ± 1.7°	2.3° ± 1.6°	0.332^a^
LTC absolute deviation	2.2° ± 1.8°	1.9° ± 1.3°	0.163^a^
HKA outlier (*n*, %)	16, 15.4%	1, 1.9%	**0.011**^b^
FFC outlier (*n*, %)	13, 12.5%	1, 1.9%	0.060^b^
FTC outlier (*n*, %)	14, 13.5%	0, 0%	**0.013**^b^
LFC outlier (*n*, %)	30, 28.8%	7, 13.5%	**0.033**^b^
LTC outlier (*n*, %)	28, 26.9%	2, 3.8%	**0.001**^b^

HKA, hip–knee–ankle angle; FFC, frontal femoral component angle; FTC, frontal tibial component angle; LFC, lateral femoral component angle; and LTC, lateral tibial component angle. Values exceeding the target value by 3 degrees were recorded as outliers

^a^Stands for *t* test, and ^b^Stands for *χ*^2^ test. *P* value in bold indicates statistical significance

The operation time, tourniquet time in the 3D preoperative planning group were significantly shorter (*P* < 0.001), while the robotics group had lower Hb loss at 1 and 3 days after operation (*P* < 0.001) (Table 3).

**Table 3:** Comparison of surgical data

	3D preoperative planning group (*n* = 104)	Robotics group (*n* = 52)	*P *value
Operation time (min)	92.2 ± 16.4	130.1 ± 26.9	**< 0.001**
Tourniquet time (min)	56.6 ± 13.5	96.1 ± 15.1	**< 0.001**
Length of hospital stay (day)	8.3 ± 1.5	8.5 ± 3.2	0.532
Hb loss 1 day (g/L)	19.5 ± 9.7	9.6 ± 9.1	**< 0.001**
Hb loss 3 days (g/L)	35.6 ± 13.9	22.9 ± 13.6	**< 0.001**

Hb, hemoglobin. The *t* test was used for all. *P* value in bold indicates statistical significance

There was no significant difference in KSS and WOMAC scores between two groups (Table 4).

**Table 4:** Comparison of patient-reported outcome measures

	3D preoperative planning group (*n* = 104)	Robotics group (*n* = 52)	*P *value
**KSS** *Knee scores (100points)*			
Pre-op	60.2 ± 18.2	61.2 ± 16.2	0.745
3 months	83.9 ± 9.3	84.4 ± 8.0	0.741
6 months	92.3 ± 9.4	93.0 ± 8.0	0.681
12 months	95.9 ± 8.8	96.8 ± 6.1	0.473
*Function scores (100points)*			
Pre-op	43.9 ± 21.2	44.6 ± 20.6	0.851
3 months	65.5 ± 10.2	66.9 ± 9.9	0.418
6 months	92.0 ± 13.2	93.4 ± 10.7	0.509
12 months	96.8 ± 14.0	98.6 ± 9.1	0.406
*Total score (200points)*			
Pre-op	104.1 ± 30.5	105.8 ± 33.1	0.758
3 months	149.4 ± 17.4	151.3 ± 15.5	0.508
6 months	184.3 ± 21.0	186.3 ± 16.7	0.546
12 months	192.6 ± 22.4	195.4 ± 14.6	0.421
**WOMAC** *Pain (100points)*			
Pre-op	63.7 ± 18.1	63.0 ± 15.7	0.807
3 months	84.8 ± 12.9	86.4 ± 11.8	0.457
6 months	91.1 ± 11.5	90.3 ± 13.6	0.712
12 months	96.2 ± 6.3	95.8 ± 7.7	0.740
*Stiffness (100points)*			
Pre-op	69.8 ± 23.7	71.2 ± 22.9	0.740
3 months	81.7 ± 12.7	81.5 ± 12.5	0.911
6 months	87.5 ± 17.2	85.6 ± 18.4	0.522
12 months	95.0 ± 9.3	95.0 ± 9.3	1.000
*Function (100points)*			
Pre-op	62.5 ± 14.9	62.1 ± 15.7	0.865
3 months	89.6 ± 8.7	89.1 ± 9.3	0.716
6 months	92.5 ± 10.7	92.2 ± 11.8	0.875
12 months	97.5 ± 5.4	97.1 ± 7.0	0.697
*Total score (100points)*			
Pre-op	65.4 ± 15.7	65.4 ± 15.0	0.984
3 months	85.4 ± 9.0	85.6 ± 9.0	0.863
6 months	90.3 ± 11.9	89.4 ± 13.5	0.639
12 months	96.2 ± 5.5	95.9 ± 6.6	0.794

KSS, Knee Society Score; WOMAC, Western Ontario and McMaster Universities Osteoarthritis Index. The *t* test was used for all

## Discussion

The most important finding of this study was that the robotics group had less malalignment, less malposition, less Hb loss, longer operative time and similar PROMs, compared with the personalized 3D preoperative planning group in TKA.

The robotics group had significantly lower FFC, FTC absolute deviation (*P* < 0.05) and less outliers in HKA, FTC, LFC and LTC (*P* < 0.05), compared with the personalized 3D preoperative planning group (Table 2). Robotics was designed for accurate alignment and prosthesis positioning, which has the advantages of intraoperative real-time navigation, secondary calibration and sensitive feedback [23–25], under the help of robotic arm and optical measurement technology. On the contrary, although there are multiple key points to reduce surgeons' subjective evaluation during intraoperative implementation under the help of 3D preoperative planning [17], arthroplasty is still partially dependent on surgeons' observation and manual operation which lead to intraoperative inaccuracy.

The Hb loss at 1 and 3 days after operation was significantly lower in robotic group (*P* < 0.001) (Table 3), and the main reason may lie in that opening of femoral medullary canal was not required under robot assistance. An RCT conducted by Kuo et al. found that avoiding opening medullary cavity could significantly reduce blood loss and transfusion rate in TKA [26]. Rathod et al. and Schnurr et al. reached the similar conclusions [27, 28].

Due to additional procedures such as preparation of robotic arms and intraoperative registration, the duration, operation and tourniquet time of robotics group were significantly longer than that of 3D preoperative planning group (92.2 min ± 16.4 min vs 130.1 min ± 26.9 min for operation time, *P* < 0.001) (Table 3). Song et al. had demonstrated that robot-assisted TKA required an additional 25 min of operation time compared with conventional TKA, even after surmounting the learning curve [29, 30]. And the previous study had shown that personalized 3D preoperative planning TKA took an average of 13 min less than conventional TKA [17]. The longer operation time of robotics group in this study was logically consistent with the above articles.

The studies conducted by Bouché et al. and Lei et al. have shown that decrease in HKA outliers was not associated with a significant improvement in the short-to-medium-term PROMs [13, 14]. On the one hand, the scoring scale nowadays may not be sensitive enough to detect potential improvements [16]. On the other hand, various factors could affect postoperative outcomes, including the target coronal alignment, soft tissue balance, rehabilitation and mental expectation. The decrease in malalignment may be more intuitive in reducing revision procedure, just as Hickey et al. projected [31], which requires a much longer follow-up.

Multiple limitations of this study must be noted before revealing the clinical relevance. Firstly, the Skywalker™ robotics system currently is exclusive for MicroPort® prosthesis. Separate MicroPort® prosthesis is not available in the authors’ hospital (not on the hospital centralized procurement list of medical supplies), which is the only prostheses that the robotics system could recognize. Although prostheses types differences had no effect on the comparison of alignment accuracy between robotics and the personalized 3D preoperative planning, this made the comparison of postoperative outcomes a little confused because various prosthesis designs might influence PROMs [32–34]. Secondly, long-term follow-up should be further explored to make this study more clinically valuable. Thirdly, being a retrospective study, although potential biases were reduced through 1:2 matching, the conclusion of this study still needs to be verified by subsequent researches.

Robotics could significantly improve alignment accuracy, but the expensive start-up costs (equipment purchase and maintenance fees, often up to $800,000 [35]) discourage many smaller-scale clinics. Similarly, excessive operating costs (advanced preoperative imaging and cleaning fees, quoted at over $1200 per case [35]) also make many patients feel overburdened, especially when the extra costs cannot be covered by medical insurance. Promoting convenience of robotics, reducing robotics-related costs and shortening operation time will provide new impetus for the development of TKA. The personalized 3D preoperative planning may be less accurate like robotics in alignment, but is much better than conventional TKA [17] with a much lower extra cost compared with robotics (no more than $280 per case). Coupled with the advantage of shorter operation time, the excellent cost performance of personalized 3D preoperative planning might make it still attractive to many surgeons.

## Conclusions

Compared with 3D preoperative planning group, patients in robotics group had significantly less malalignment, malposition, Hb loss, but similar PROMs. The operations in robotics group spent longer operation time and cost more compared with 3D preoperative planning group.

## Supplementary Information

**Additional file 1:** Detailed procedure of the personalized 3D preoperative planning.

## Data Availability

All data and materials of the present study were in full compliance with the journal’s policy.

## Abbreviations

TKA: Total knee arthroplasty
3D: Three-dimensional
CT: Computed tomography
FLX: Full-length radiograph
PSI: Patient-specific instrumentation
HKA: Hip–knee–ankle angle
FFC: Frontal femoral component angle
FTC: Frontal tibial component angle
LFC: Lateral femoral component angle
LTC: Lateral tibial component angle
ICC: Intraclass correlation coefficient
BMI: Body mass index
Hb: Hemoglobin
RCT: Randomized controlled trial
PSM: Propensity score matching
