# Supplementary material for: Robotics versus personalized 3D preoperative planning in total knee arthroplasty: a propensity score-matched analysis
Source: J Orthop Surg Res. 2022 Apr 11;17:227. doi: 10.1186/s13018-022-03115-3 (PMC8996413; doi:10.1186/s13018-022-03115-3)
Supplement: Supplementary file 1 — Additional file 1: Detailed procedure of the personalized 3D preoperative planning. [file 13018_2022_3115_MOESM1_ESM.docx]

**Appendix** Detailed procedure of the personalized 3D preoperative planning

The authors used the full-length Computed Tomography (CT) data of the patients (thin scan of 1 mm at the knee joint and thick scan of 3 mm at the rest parts) and performed 3D reconstruction with Mimics Research 19.0. With the CATIA 5.20 and NX12.0 software, the engineer and the surgeon at the authors’ Center formulated the personalized 3D preoperative planning. The best spherical fitting of the femoral head was performed, and the line between the obtained center point and the apex of the femoral intercondylar notch was defined as the mechanical axis of the femur; the line connecting the center points of femoral medullary cavity 10 cm and 20 cm above the knee joint line was defined as the anatomical axis of the distal femur[1, 2]; the line between the most prominent point of the lateral femoral epicondyle and the most concave point of the medial femoral epicondyle was the surgical trans-epicondylar axis (sTEA)[3]. The plane defined by the femoral mechanical axis and sTEA was recorded as the femoral coronal plane (the femoral mechanical axis was in this coronal plane, and sTEA was parallel to the coronal plane); the plane perpendicular to the femoral mechanical axis was recorded as the transverse plane; the line connecting the center points of tibial medullary cavity at 5 cm below the tibial tubercle and 5 cm above the ankle mortise was defined as the tibial anatomical axis. In the preoperative design, the key information needed to be obtained were: 1) the projection angle of the HKS on the coronal plane (Fig. 1); 2) the position where the distal femoral anatomical axis penetrated the distal femur, which was recorded as the femoral entry point(Fig.2A); 3) the projection angle of the sTEA and the posterior femoral condylar tangents on the transverse plane, which was recorded as the posterior condylar angle(PCA); 4) the position where the tibial anatomical axis penetrated the tibial plateau, which was recorded as the fix point of the tibial plateau extramedullary guide pin(Fig.2D); 5) distance between the midpoint of the ankle mortise and the midpoint of medial and lateral malleolus(Fig.2H); 6) thickness of the anterior tibial soft tissue at 3 cm above the ankle mortise(Fig.2K) and the distance between the anterior tibial cortex and the distal tibial extramedullary positioning rod(Fig.2L); 7) the volume of femoral and tibial osteotomy. During the surgery, the femoral entry point was selected strictly according to the preoperative plan (Fig.2A, B); the osteotomy angle was selected based on the projection angle of the HKS on the coronal plane (Fig.2C), and the distal osteotomy was guided according to the specific osteotomy volume. Based on the preoperative plan, the fix point of the tibial plateau extramedullary guide pin was selected to determine the proximal end of the extramedullary positioning rod (Fig.2D-G). The thickness of the anterior tibial soft tissue at 3 cm above the ankle mortise (Fig.2K), the distance between the anterior tibial cortex and the distal tibial extramedullary positioning rod (Fig.2L) as well as the distance between the midpoint of the ankle mortise and the midpoint of medial and lateral malleolus (Fig.2H) were used to determine the distal end of the rod (Fig.2H-N). The tibial osteotomy was conducted according to the osteotomy volume. The femoral rotatory osteotomy was conducted referring to the PCA. The remaining surgical techniques were the same as the conventional TKA.


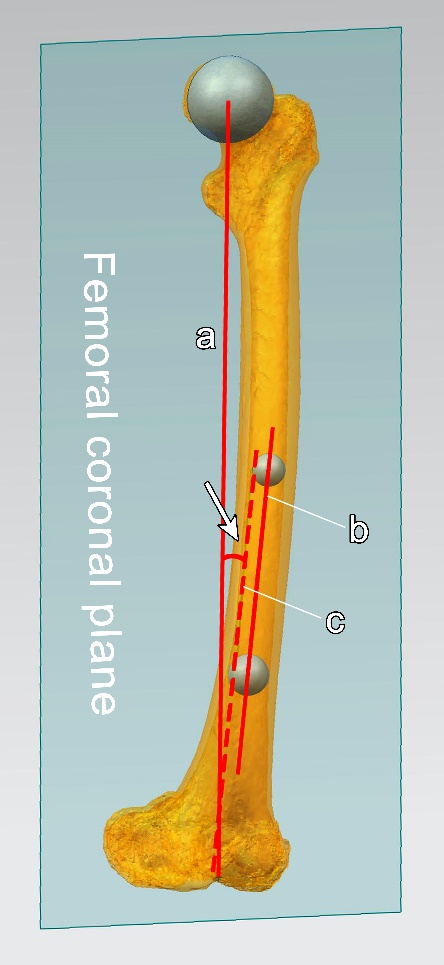


**Figure 1** The projection angle of the Hip-Knee-Shaft on the coronal plane

The blue plane in the figure was the femoral coronal plane (the femoral mechanical axis was in this coronal plane, and the surgical trans-epicondylar axis was parallel to the coronal plane); **line** **a** was the femoral mechanical axis, **line** **b** was the anatomical axis of the distal femur, and line **c** was the coronal projection of the distal femoral anatomical axis; the angle between **line a** and **line c** is the projection angle of the HKS on the coronal plane.

**
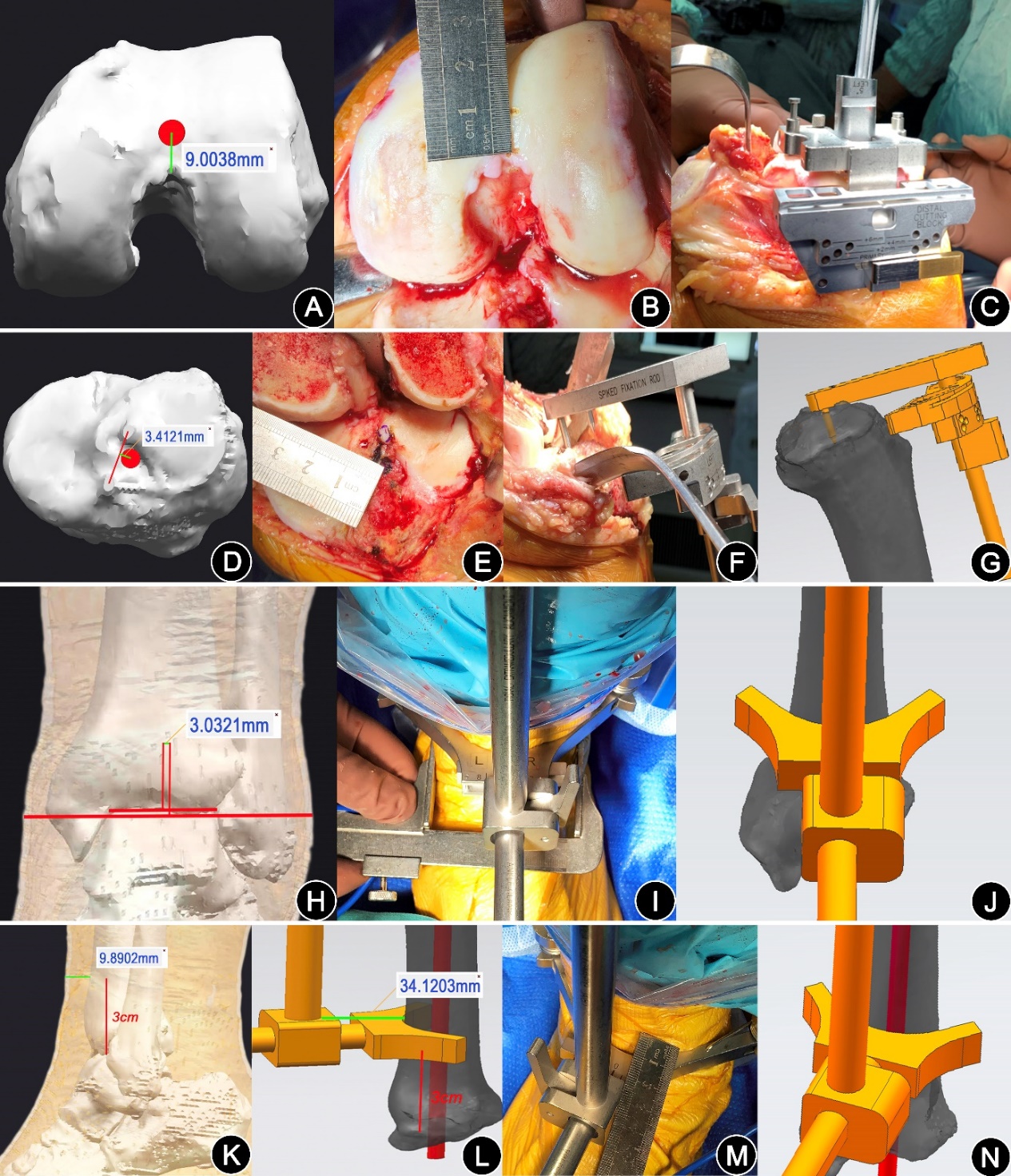
**

**Figure 2** Illustration of the key steps of the personalized 3D preoperative planning.

***(Fig.2A-C)*** The distal femoral osteotomy. The red dot in *Fig.2A* was the position where the anatomical axis of the distal femur penetrated the distal femur cortex on the preoperative 3D model, i.e., the femoral entry point; *Fig.2B* showed the accurate positioning of the femoral entry point during surgery; *Fig.2C* showed the intramedullary positioning rod was inserted from the femoral entry point into the medullary cavity for about 20 cm, and the distal femur osteotomy was performed according to the projection angle of the HKS on the coronal plane.

***(Fig.2D-G)*** showed the positioning of the proximal end of the tibial extramedullary rod. The red dot in *Fig.2D* was the position where the tibial anatomical axis penetrated the tibial plateau cortex, i.e., the fix point of the tibial plateau extramedullary guide pin; *Fig.2E* showed the accurate positioning of the above anchor point during surgery; *Fig.2F* and *Fig.2G* showed that the tibial extramedullary guide pin was fixed to the anchor point to ensure the accurate positioning of the extramedullary positioning rod at the proximal end of the tibia during the surgery and on the preoperative 3D models, respectively.

***(Fig.2H-J)*** showed the coronal positioning of the distal end of the tibial extramedullary rod; *Fig.2H* showed distance between the midpoint of the ankle mortise and the midpoint of medial and lateral malleolus; *Fig.2I* and *Fig.2J* showed the accurate positioning of the distal end of the tibial extramedullary rod on the coronal plane with reference to the above preoperative design during the surgery and on the preoperative 3D model, respectively.

***(Fig.2K-N)*** showed the sagittal positioning of the distal end of the tibial extramedullary rod. *Fig.2K* showed the thickness of the anterior tibial soft tissue at 3 cm above the ankle mortise; *Fig.2L* showed the distance between the anterior tibial cortex and the distal tibial extramedullary positioning rod; *Fig.2M* and *Fig.2N* showed the accurate positioning of the distal end of the tibial extramedullary rod on the sagittal plane was ensured with reference to the distance between the anterior tibial skin and the distal tibial extramedullary positioning rod(the distance between the anterior tibial cortex and the distal tibial extramedullary positioning rod minus the thickness of the anterior tibial soft tissue) during the surgery and on the preoperative 3D model, respectively.

**References of Appendix**

1. Lasam MP, Lee KJ, Chang CB, Kang YG, Kim TK (2013) Femoral lateral bowing and varus condylar orientation are prevalent and affect axial alignment of TKA in Koreans. Clin Orthop Relat Res 471:1472-1483. DOI 10.1007/s11999-012-2618-7

2. Liu T, Wang CY, Xiao JL, Zhu LY, Li XZ, Qin YG, Gao ZL (2014) Three-dimensional reconstruction method for measuring the knee valgus angle of the femur in northern Chinese adults. J Zhejiang Univ Sci B 15:720-726. DOI 10.1631/jzus.B1400019

3. Boisgard S, Moreau PE, Descamps S, Courtalhiac C, Silbert H, Moreel P, Michel JL, Levai JP (2003) Computed tomographic study of the posterior condylar angle in arthritic knees: its use in the rotational positioning of the femoral implant of total knee prostheses. Surg Radiol Anat 25:330-334. DOI 10.1007/s00276-003-0144-8
